# Concentrations of bile acid precursors in cerebrospinal fluid of Alzheimer's disease patients

**DOI:** 10.1016/j.freeradbiomed.2018.12.020

**Published:** 2019-04

**Authors:** William J. Griffiths, Jonas Abdel-Khalik, Eylan Yutuc, Gustavo Roman, Margaret Warner, Jan-Åke Gustafsson, Yuqin Wang

**Affiliations:** a Swansea University Medical School, ILS1 Building, Singleton Park, Swansea SA2 8PP, UK; b Methodist Neurological Institute, Methodist Hospital, Houston, TX 77030, USA; c Department of Biology and Biochemistry, Center for Nuclear Receptors and Cell Signaling, University of Houston, 3517 Cullen Blvd, Houston, TX 77204, USA

**Keywords:** Sterol, Cholesterol, Oxysterol, Cholestenoic acid, Bile acid, Brain, Neurodegenerative disease, Mass spectrometry, Cytochrome P450

## Abstract

Using liquid chromatography – mass spectrometry in combination with derivatisation chemistry we profiled the oxysterol and cholestenoic acid content of cerebrospinal fluid from patients with Alzheimer's disease (n = 21), vascular dementia (n = 11), other neurodegenerative diseases (n = 15, Lewy bodies dementia, n = 3, Frontotemporal dementia, n = 11) and controls (n = 15). Thirty different sterols were quantified and the bile acid precursor 7α,25-dihydroxy-3-oxocholest-4-en-26-oic acid found to be reduced in abundance in cerebrospinal fluid of Alzheimer's disease patient-group. This was the only sterol found to be changed amongst the different groups.

## Highlights

• More than 30 sterols/oxysterols/sterol-acids analysed in human CSF.
• 7α,25-Dihydroxy-3-oxocholest-4-en-26-oate reduced in AD patient-group.
• 7α,25-Dihydroxy-3-oxocholest-4-en-26-oate – an intermediate in bile acid synthesis.
• Bile acid synthesis linked to AD.

## Graphical abstract

**Figure:**
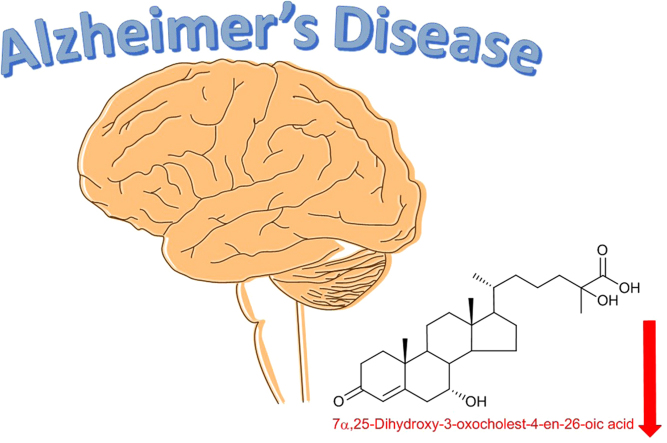

## 1 Introduction

Cholesterol has been linked to the aetiology of Alzheimer's disease (AD) for decades with the ε4 allele of apolipoprotein E gene (*APOE*) being the most robust genetic risk factor for sporadic AD [1], [2]. More recently cholesterol metabolism-related genes including *ABCA7* (ATP binding cassette subfamily A member 7), *ABCG1* (ATP binding cassette subfamily G member 1), *CLU* (apolipoprotein J) and *SORL1* (LDLR relative with 11 ligand-binding repeats) have been classified among susceptibility loci by large genome-wide association studies (GWAS) [3], [4]. Furthermore, in 2018 Picard et al. reported that polymorphism rs2269657 of the *SREBF2* gene, which codes for the protein sterol regulatory element-binding protein-2 (SREBP-2), the master transcription factor regulating cholesterol biosynthesis, showed significant dual association with late-onset AD pathological biomarkers and gene expression levels [5]. Expression levels of the rs2269657 allele of *SERBF2* in frontal cortex from late-onset AD brain inversely correlated with plaque density and with age at death [5].

Cholesterol is abundant in brain (2% wet weight) with about 25% of the total body cholesterol being found in brain [6]. Cholesterol cannot cross the blood brain barrier (BBB), hence after parturition essentially all cholesterol in brain is synthesised in situ from acetyl CoA, and all cholesterol export is via metabolism [6]. The first step of cholesterol metabolism is formation of an oxysterol, an oxidised form of cholesterol, and subsequent metabolism leads to steroid hormones and bile acids [7], i.e. in the central nervous system (CNS) to neurosteroids and C_27_ bile acids [8], [9]. In brain the dominant oxysterol is 24S-hydroxycholesterol [10], formed in neurons by oxidation of cholesterol by CYP46A1 (cytochrome P450 family 46 subfamily A member 1) [11], this can be metabolised further in CNS [12] or exported as the intact molecule over the BBB [13]. Björkhem and colleagues have suggested the balance between 24S-hydroxycholesterol and its positional isomer (25 R)26-hydroxycholesterol (common name 27-hydroxycholesterol) in brain may affect the production of beta-amyloid in brain [14]. (25R)26-Hydroxycholesterol may be formed via CYP27A1 (cytochrome P450 family 27 subfamily A member 1) mediated oxidation of cholesterol in brain or imported into brain from extracerebral sources [14], [15], [16]. (25R)26-Hydroxycholesterol is elevated in AD brain [17] and Björkhem et al. have suggested (25R)26-hydroxycholesterol may provide a missing link between hypercholesterolemia and AD [14].

While it is difficult to investigate brain from living subjects, cerebrospinal fluid (CSF), the fluid which bathes the brain, is available. Papassotiropoulos et al. and Schönknecht et al. found elevated CSF concentrations of 24S-hydroxycholesterol in AD patients, which they explained by increased cholesterol turnover during neurodegeneration [18], [19]. Surprisingly, this elevation is not evident in AD plasma [14]. CYP46A1 is normally expressed in neurons [11], but in AD is also expressed in glia cells [20]. 24S-Hydroxycholesterol reduces expression of enzymes of the cholesterol biosynthesis pathway in mouse neurons and glia, presumably by inhibiting the processing of SREBP-2 to its active form as a transcription factor, but up-regulates expression of APOE [21], [22]. *ApoE* is a target gene of liver X receptors (LXRα and β, NR1H3 and NR1H2), both of which are expressed in mouse brain [23], and known to be activated by oxysterols, including 24S-hydroxycholesterol [24]. (25 R)26-Hydroxycholesterol has also been found to be elevated in CSF from AD patients [25], [26].

The end products of cholesterol metabolism include bile acids and steroid hormones. Intermediates in the bile acid biosynthesis pathways have been observed in human and rodent CSF [8], [9], [12], [27], [28] and brain [29], [30], while bile acids have been found in rodent [29], [31], [32] and human brain [33]. Pan et al. found levels of taurocholic acid were reduced in AD brain [33]. When plasma was analysed they found cholic acid to be reduced in AD patients [33]. Others have found lithocholic acid to be increased in plasma from AD patients [34], while in an untargeted metabolomics study the glycine conjugates of cholic acid, deoxycholic acid and chenodeoxycholic acid have been found to be elevated in AD plasma [35].

In an effort to understand further the relationship between cholesterol metabolism and AD we have profiled the oxysterol content of CSF from AD patients, those with vascular dementia (VD) and other neurodegenerative disease (OND), and normal controls with no evidence of “normal pressure hydrocephalus” (NPH). We have extended the profile to include cholesterol and its immediate precursors and intermediates in the acidic-, neutral-, 24S-hydroxylase- and 25-hydroxylase-pathways of bile acid biosynthesis. Of the 30 sterols routinely identified in CSF, between the different patient/control groups, only one was changed in abundance in CSF, whether normalised to the cholesterol content (ng/µg-cholesterol) or measured in ng/mL, this was the bile acid precursor 7α,25-dihydroxy-3-oxocholest-4-en-26-oic acid which was reduced in concentration in CSF from the AD group.

## 2 Methods

### 2.1 Biomaterial

CSF samples were obtained from patients evaluated at the Memory Clinic, Methodist Hospital, Houston, because of complaints of memory loss. They were all found to have enlarged ventricles and were considered “probable NPH”. A spinal tap was performed to confirm or not the diagnosis of NPH. After the spinal tap with removal of CSF (material analysed in the current study) the following diagnostic categories were concluded: (i) No evidence of NPH (these are considered “normal” controls, n = 15); cases of NPH plus (ii) AD, n = 21, (iii) OND, n = 15, including Lewy bodies dementia, LBD, n = 3; Frontotemporal Dementia, FTD, n = 11) and Vascular Dementia (VD, n = 11, See Supplemental Table S1 for diagnostic information). All the CSF samples were obtained with a similar technique under fluoroscopy by a hospital neuroradiologist. Participants in the study gave written, informed consent to participate in the study, which was conducted according to the Declaration of Helsinki and its subsequent amendments.

### 2.2 Methods

Sterols including oxysterols and bile acid precursors were analysed by liquid chromatography (LC) – mass spectrometry (MS) incorporating charge-tagging methodology, termed “Enzyme-Assisted Derivatization for Sterol Analysis” (EADSA) [9], [36], [37]. The method is fully described in Abdel-Khalik et al. [9]. In brief, sterols were extracted into ethanol from CSF, oxidised with cholesterol oxidase and derivatized with deuterated Girard P reagent ([^2^H_5_]GP). A separate aliquot of extract was similarly derivatized with [^2^H_0_]GP in the absence of enzyme (Supplemental Fig. S1). The derivatives were combined and analysed by LC-MS, exploiting high mass-resolution (120,000, full-width at half-maximum height definition) and multistage fragmentation (MS^n^). Quantification was achieved, where possible, by the addition of isotope-labelled standards. In the absence of an exact isotope-labelled standard quantification was made against isotope-labelled structural-analogues (See Supplemental Tables S2–S4) [9], [37]. Crick et al. and Karu et al. have previously demonstrated the efficiency of the extraction, derivatisation and purification methods and Crick et al. validated the method for multiple sterols and C_27_ bile acids [37], [38]. Here, despite the absence of an authentic isotope-labelled standard for 7α,25-dihydroxy-3-oxocholest-4-en-26-oic acid we have confirmed the validity of using [^2^H_7_]24(R/S)-hydroxycholesterol as its internal standard by the standards addition method, adding consecutively increasing amounts of acid (0.2 ng/mL – 0.8 ng/mL) to a pooled CSF sample containing a set concentration of [^2^H_7_]24(R/S)-hydroxycholesterol (8 ng/mL).

### 2.3 Statistics

An ANOVA was run against each sterol. Univariate *t*-tests were performed against the control group. P < 0.05 (*) is considered statistically significant. Concentrations in the text are given in units of ng/mL or ng/µg-cholesterol as mean ± standard deviation (SD).

## 3 Results

In the current study we measured the concentrations of unesterified “free” sterols, oxysterols and bile acid precursors in the absence of saponification or solvolysis. This contrasts to most other studies of sterols and oxysterols by other groups where a solvolysis step is included to hydrolyse fatty acid esters [18], [19], [25]. Data for the control sample set has been reported previously in [9]. In the absence of authentic isotope-labelled standards for cholestenoic acids, [^2^H_7_]24(R/S)-hydroxycholesterol was used as the internal standard. This selection has previously been validated by Crick et at for some C_27_ acids [37] and here specifically for 7α,25-dihydroxy-3-oxocholest-4-en-26-oic acid using the standard additions method where a plot of measured concentration against theoretical concentration gave a straight line with R^2^ > 0.994.

### 3.1 Oxysterols in CSF

**Fig. 1:**
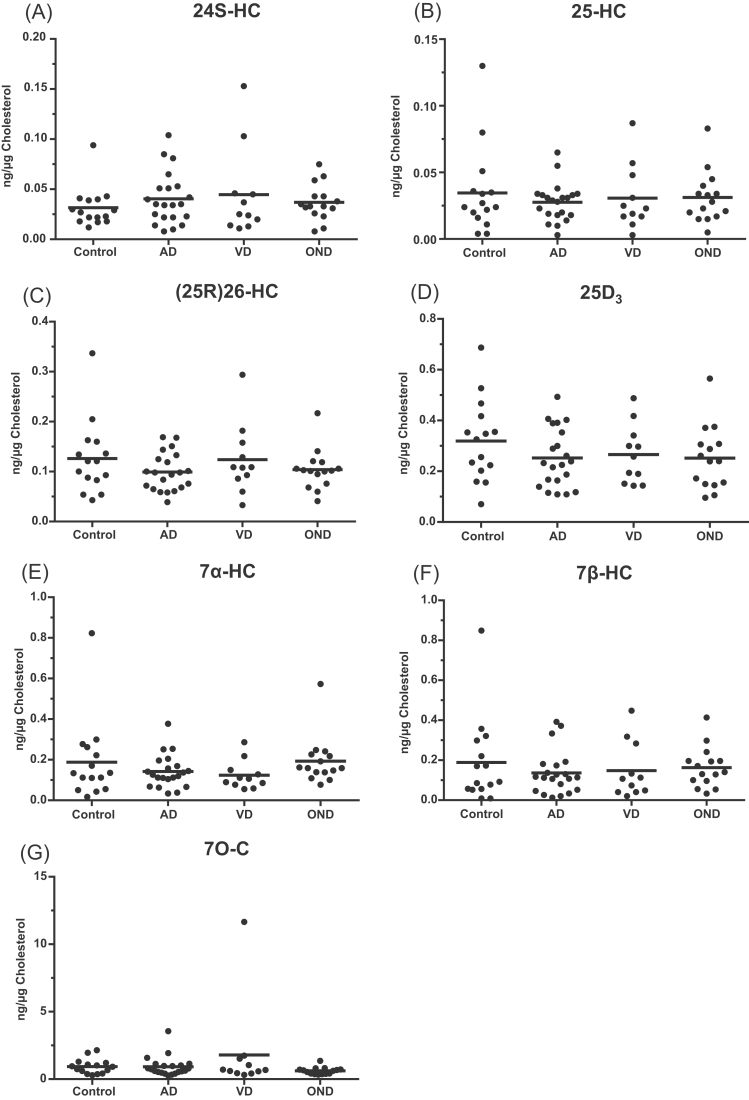
Dot-plots displaying the concentrations of different oxysterols in CSF. Each dot indicates an individual patient sample. Concentrations are in ng/µg-cholesterol. The black bars indicate the mean value for each group. There were no statistical differences between the disease and control groups. (A) 24S-HC, 24S-hydroxycholesterol; (B) 25-HC, 25-hydroxycholesterol; (C) (25 R)26-HC, (25 R)26-hydroxycholesterol; (D) 25D_3_, 25-hydroxyvitamin D_3_; (E) 7α-HC, 7α-hydroxycholesterol; (F) 7β-HC, 7β-hydroxycholesterol; (G) 7O-C, 7-oxocholesterol. Abbreviations: - AD, Alzheimer's disease; VD, vascular dementia; OND, other neurodegenerative diseases (i.e. Lewy bodies dementia, Frontotemporal dementia).

**Fig. 2:**
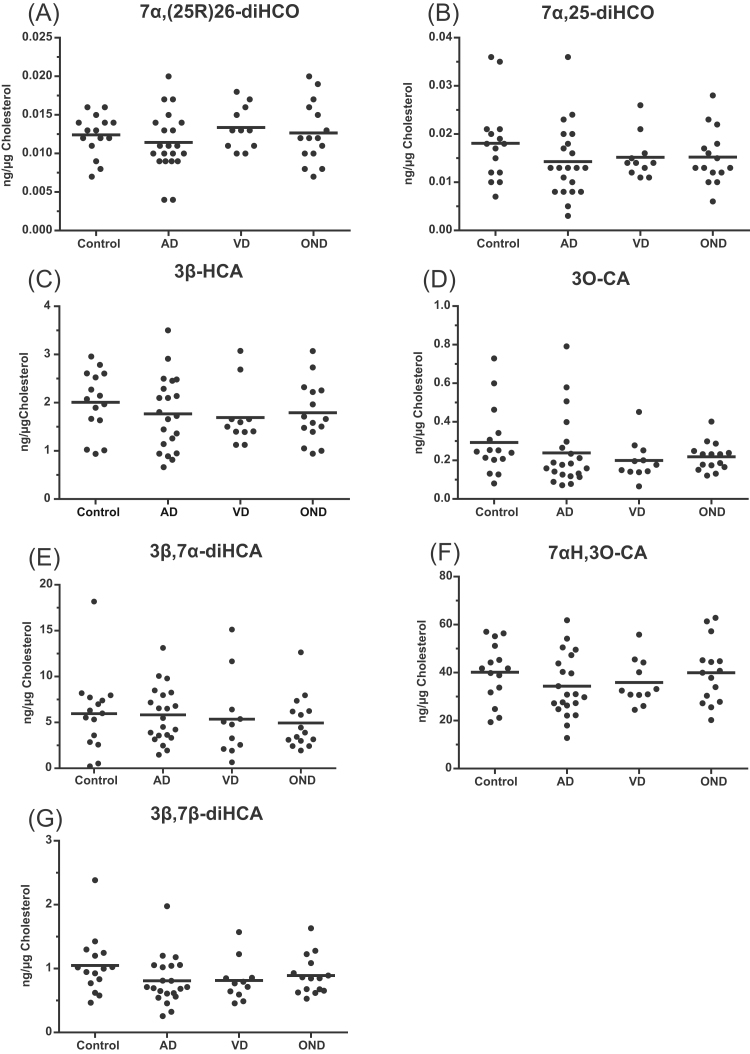
Dot-plots displaying the concentrations of different dihydroxysterols and cholestenoic acids in CSF. Each dot indicates an individual patient sample. Concentrations are in ng/µg-cholesterol. The black bar indicates the mean value. (A) 7α,(25 R)26-diHCO, 7α,(25 R)26-dihydroxycholest-4-en-3-one; (B) 7α,25-diHCO, 7α,25-dihydroxycholest-4-en-3-one; (C) 3β-HCA, 3β-hydroxycholest-5-en-(25 R)26-oic acid; (D) 3O-CA, 3-oxocholest-4-en-(25 R)26-oic acid; (E) 3β,7α-diHCA, 3β,7α-dihydroxycholest-5-en-(25 R)26-oic acid; (F) 7αH,3O-CA, 7α-hydroxy-3-oxocholest-4-en-(25 R)26-oic acid; (G) 3β,7β-diHCA, 3β,7β-dihydroxycholest-5-en-(25 R)26-oic acid.

The concentrations of monohydroxycholesterols, dihydroxycholesterols and dihydroxycholest-4-en-3-ones in CSF are low, ranging from about the limit of quantification of the method, 0.01 ng/mL, for the dihydroxysterols to about 0.1 ng/mL for (25 R)26-hydroxycholesterol and 7β-hydroxycholesterol (Supplemental Tables S2 and S3). There was no statistically significant difference in concentration measured, as either ng/mL or ng/µg-cholesterol, in any of the oxysterols investigated between the different sample groups ( Figs. 1 and 2, Supplemental Fig. S2).

### 3.2 Bile acid precursors in CSF

As in other studies [8], [9], [12], [27], [28], we measured intermediates of the acidic pathway of bile acid biosynthesis in CSF (Supplemental Fig. S3). These include 3β-hydroxycholest-5-en-(25 R)26-oic acid, its CYP7B1 (cytochrome P450 family 7 subfamily B member 1) metabolite, 3β,7α-dihydroxycholest-5-en-(25 R)26-oic acid and the 3-oxo metabolite, 7α-hydroxy-3-oxocholest-4-en-(25 R)26-oic acid, formed by oxidation at C-3 and Δ^5^-Δ^4^ isomerisation by HSD3B7 (3beta-hydroxysteroid dehydrogenase type 7). The latter compound is present at high levels (~28 ng/mL, ~40 ng/µg-cholesterol) in CSF (Fig. 2, see also Supplemental Fig. S4 and Tables S2 & S3). 7α-Hydroxy-3-oxocholest-4-en-(25 R)26-oic acid can be metabolised in the peroxisome to the Co-A thioesters of 7α,24R-dihydroxy-3-oxocholest-4-en-(25 R)26-oic acid, then 7α-hydroxy-3,24-*bis*oxocholest-4-en-(25 R)26-oic acid and ultimately 7α-hydroxy-3-oxochol-4-en-24-oic acid [30], [39]. In our assay we observe the hydrolysed thioesters, but, in the absence of authentic standards of the different diasteriomers, could not determine the stereochemistry at C-25. 7α,24-Dihydroxy-3-oxocholest-4-en-26-oic acid could also be derived from 24S-hydroxycholesterol and have 24S-,25R- or 24S-,25S-stereochemistry [12].

**Fig. 3:**
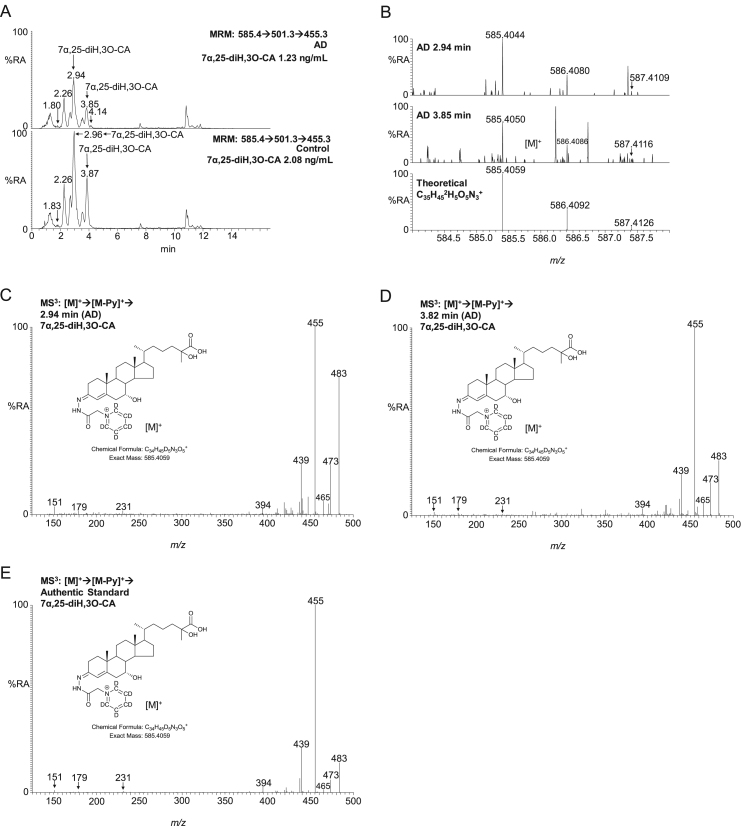
(A) Multiple reaction monitoring (MRM) chromatograms corresponding to the transitions *m/z* 585.4→501.3→455.3 targeting [^2^H_5_]GP-derivatised dihydroxy-3-oxocholest-4-en-26-oic acids. The transition is particularly prominent in 7α,25-dihydroxy-3-oxocholest-4-en-26-oic acid (7α,25-diH,3O-CA) [12]. GP-derivatives appear as twin peaks as a consequence of the formation of *syn* and *anti* conformers of the derivative. The upper panel displays a chromatogram from AD CSF, the lower panel from control CSF. The measured concentrations (ng/mL) of 7α,25-diH,3O-CA are given in the right-hand corners of the chromatograms. (B) High-resolution mass spectra of the *syn* and *anti* conformers of 7α,25-diH,3O-CA eluting at 2.94 min (upper panel) and 3.85 min (central panel) from the AD CSF sample and the theoretical isotope pattern for their chemical formula C_35_H_45_^2^H_5_O_5_N_3_^+^. MS^3^ spectra [M]^+^→[M-Py]^+^→ (*m/z* 585.4→501.3→), where Py is pyridine, from the conformers eluting at (C) 2.94 min (D) 3.82 min from the AD sample and (E) the authentic standard of 7α,25(R/S)-dihydroxy-3-oxocholest-4-en-26-oic acid. The high-resolution mass spectra shown in (B) were recorded in the Orbitrap analyser, other data was generated in the linear ion-trap of the Orbitrap Elite mass spectrometer.

**Fig. 4:**
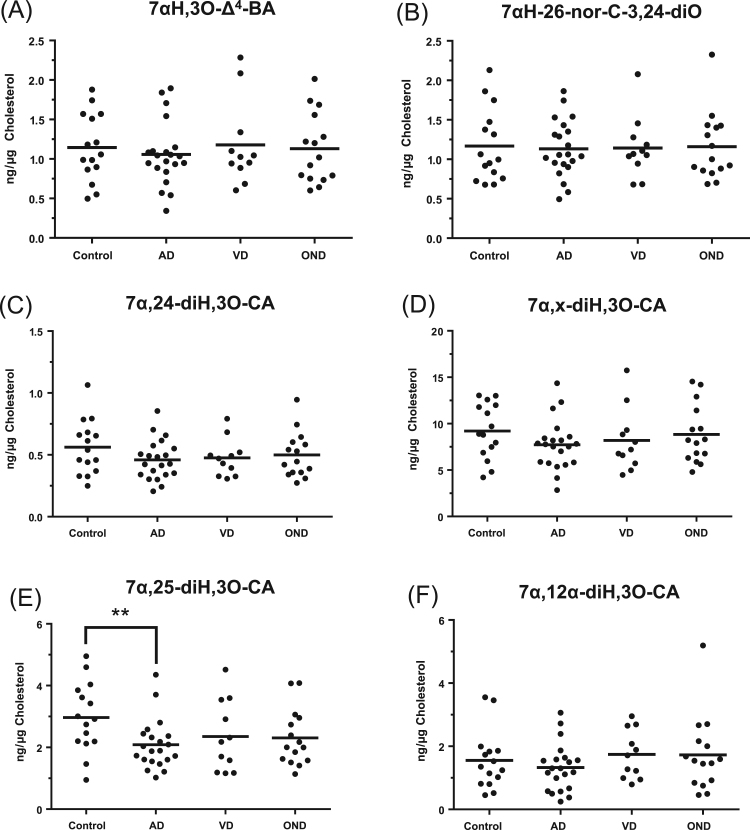
Dot-plots displaying the concentrations of different dihydroxy-3-oxocholest-4-en-26-oic and chol-4-en-24-oic acids in CSF. Each dot indicates an individual patient sample. Concentrations are in ng/µg-cholesterol. The black bar indicates the mean value. Only 7α,25-dihydroxy-3-oxocholest-4-en-26-oic acid showed a statistical difference between disease and control groups. (A) 7αH,3O-Δ^4^-BA, 7α-hydroxy-3-oxochol-4-en-24-oic acid; (B) 7αH-26-nor-C-3,24-diO, 7α-hydroxy-26-*nor*-cholest-4-en-3,24-dione; (C) 7α,24-diH,3O-CA, 7α,24-dihydroxy-3-oxocholest-4-en-26-oic acid; (D) 7α,x-diH,3O-CA, 7α,x-dihydroxy-3-oxocholest-4-en-26-oic acid; (E) 7α,25-diH,3O-CA, 7α,25-dihydroxy-3-oxocholest-4-en-26-oic acid; (F) 7α,12α-diH,3O-CA, 7α,12α-dihydroxy-3-oxocholest-4-en-26-oic acid. 7α-Hydroxy-26-*nor*-cholest-4-en-3,24-dione is formed by decarboxylation of 7α-hydroxy-3,24-*bis*oxocholest-4-en-26-oic acid [8]. The location of the second hydroxy group, indicated by x, in 7α,x-diH,3O-CA is on the C_17_ side-chain, probably at C-22 or C-23 [12].

A bile acid precursor prevalent in CSF is 7α,25-dihydroxy-3-oxocholest-4-en-26-oic acid (Fig. 3). The identity of this metabolite was confirmed by comparison of retention time, exact mass and MS^3^ spectra to that of the authentic standard 7α,25(R/S)-dihydroxy-3-oxocholest-4-en-26-oic acid. The concentration of 7α,25-dihydroxy-3-oxocholest-4-en-26-oic acid falls in CSF from 2.04 ± 0.61 ng/mL in the control group to 1.63 ± 0.52 ng/mL in the AD group (Supplemental Fig. S5). This difference is statistically significant (P < 0.05). When measured in ng/µg-cholesterol the control group and AD group concentrations are 2.97 ± 1.12 and 2.09 ± 0.8, respectively, in which case P < 0.01 (Fig. 4). Of all the bile acid precursors this was the only one found to be changed significantly in any of the patient-groups.

### 3.3 Cholesterol

**Fig. 5:**
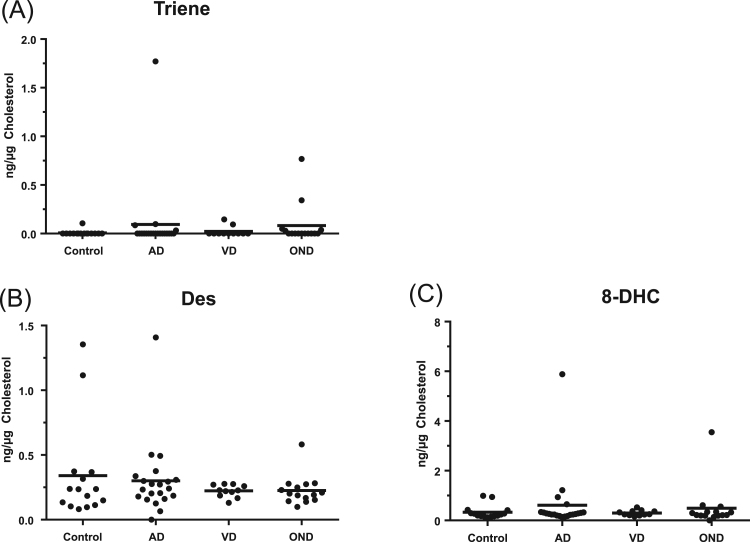
Dot-plots displaying the concentrations of a cholestatrien-3β-ol, desmosterol and 8-dehydrocholesterol in CSF. Each dot indicates an individual patient sample. Concentrations are in ng/µg-cholesterol. The black bar indicates the mean value. (A) Triene, cholestatrien-3β-ol; (B) Des, desmosterol; (C) 8-DHC, 8-dehydrocholesterol.

We also measured the levels of cholesterol and its precursors desmosterol and 7-dehydrocholesterol and the isomer 8-dehydrocholesterol. There was no statistical difference in concentrations of these sterols between the different sample groups (Fig. 5 and Supplemental Fig. S6 and Table S4).

### 3.4 Pairwise correlation between CSF levels of analytes

**Fig. 6:**
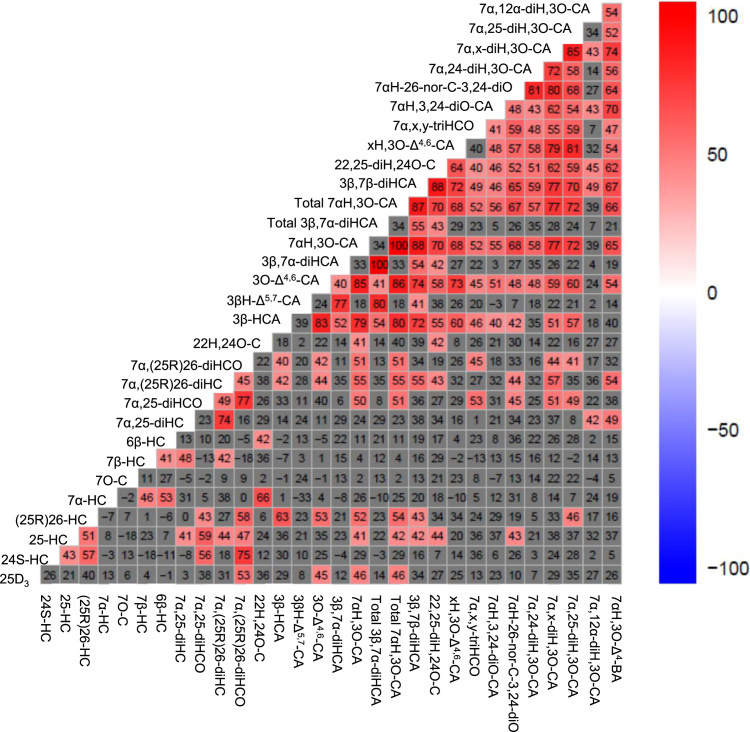
Heat map generated using the program R version 3.2.2 displaying pairwise correlations between cholesterol metabolite concentrations in human CSF samples. Values in the heat map are Spearman's rank correlation coefficients (r) × 100 between the indicated pairs of metabolites. A bipolar gradient between red (positive correlation) and blue (negative correlation) is used. Only values greater or lesser than ± 40 show statistically significant correlation at a 1% significance level. Values within ± 40 have been marked grey to show that these correlations are not statistically significant. Abbreviations for cholesterol metabolites are as in Figure Captions 1 – 5 and Supplemental Table S2.

Spearman's rank correlation can aid the determination of whether the levels of two analytes correlate, either positively or negatively. The higher or lower the statistical score for the correlation on a scale from + 100 to −100, the more likely it is that the analytes correlate positively or negatively. Scores are called the correlation coefficients (r) [40], [41]. Shown in Fig. 6 is a heat map of Spearman's rank correlation coefficients (r) × 100 between the indicated pairs of analytes. A bipolar gradient between red (positive correlation) and blue (negative correlation) is indicated by the scale on the righthand side of the Figure. Only values greater than ± 40 show statistically significant correlation at a 1% significance level. Values lower than ± 40 have been marked grey to show that these correlations are not statistically significant. Perhaps not surprisingly, 7α,25-dihydroxy-3-oxocholest-4-en-26-oic acid positively correlates most strongly with other cholestenoic acids and the C_24_ acid 7α-hydroxy-3-oxochol-4-en-24-oic acid. Interestingly, it also correlates positively with 7α,(25 R)26-dihydroxycholest-4-en-3-one, 7α,25-dihydroxycholest-4-en-3-one and (25 R)26-hydroxycholesterol.

## 4 Discussion

A limitation of the present study is a lack of isotope-labelled authentic standards for many of the metabolites studied, necessitating the use of structural analogues (See Supplemental Tables S2–S4). Earlier studies by Crick et al. have indicated that this is a valid approach when incorporating GP-derivatisation which equalises for structure-specific variation in ionisation efficiency by incorporating a permanent positive-charge in the analyte [37]. Crick et al. have also shown that in the absence of isotope-labelled standards correction for analyte loss during sample preparation can made by the use of standards with similar hydrophobicity [37]. Here we have confirmed this for 7α,25-dihydroxy-3-oxocholest-4-en-26-oic acid by performing a standard additions experiment, where R^2^ for the plot of measured concentration against theoretical concentration was found to be > 0.994.

**Fig. 7:**
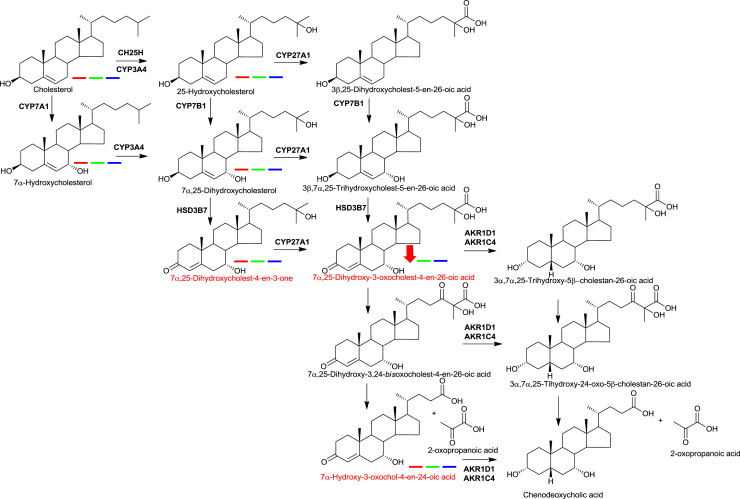
Potential routes of biosynthesis and metabolism of 7α,25-dihydroxy-3-oxocholest-4-en-26-oic acid in the CNS. Coloured bars indicate metabolites detected but not changed in concentration, while coloured arrows indicate metabolites changed in concentration. Red, green and blue correspond to Alzheimer's disease, vascular dementia or other neurodegenerative disease, respectively. Metabolites, named by red text show significant Spearman's rank correlation coefficients to 7α,25-dihydroxy-3-oxocholest-4-en-26-oic acid. Abbreviations: CYP, cytochrome P450; HSD, hydroxysteroid dehydrogenase; AKR, aldo-keto reductase.

We have previously reported the presence of 7α,25-dihydroxy-3-oxocholest-4-en-26-oic acid in human CSF [9], [12], where, as here, the identification was made based on retention time, exact mass and MS^3^ spectra of the GP-derivative and comparison to the authentic standard which was available as a mixture of 25R- and 25S-epimers. In the present study, and those made earlier, we cannot be sure of the exact stereochemistry at C-25 of the acid found in CSF. The origin of 7α,25-dihydroxy-3-oxocholest-4-en-26-oic acid is possibly via CYP3A4 (cytochrome P450 family 3 subfamily A member 4) catalysed 25-hydroxylation after 7α-hydroxylation and before, or after, C-26-hydroxylation and -carboxylation by CYP27A1 (Fig. 7, see also Supplemental Fig. S7) [12]. This would be consistent with 7α,25-dihydroxy-3-oxocholest-4-en-26-oic acid correlating positively with 7α,25-dihydroxycholest-4-en-3-one. We have demonstrated that CYP3A4 hydroxylates 7α-hydroxycholesterol [42], while Honda et al. have shown that CYP3A4, like CH25H (cholesterol 25-hydroxylase), is a 25-hydroxylase [43]. Alternatively, the start of the biosynthesis pathway for 7α,25-dihydroxy-3-oxocholest-4-en-26-oic acid may be 25-hydroxylation of cholesterol by CH25H [44].

The correlation data presented in Fig. 6 is consistent with 7α,25-dihydroxy-3-oxocholest-4-en-26-oic acid being formed via the enzymes CYP3A4, CYP27A1, HSD3B7 and either CYP7A1 or CYP7B1 (Fig. 7, Supplemental Fig. S7). With the exception of CYP7A1, each of these enzymes is known to be expressed in human brain [8], [45], [46], [47], [48], [49], while 7α-hydroxycholesterol is known to be present in rodent brain, presumably originating in the periphery and crossing the BBB into brain or being derived from non-enzymatic oxidation of cholesterol in brain [42]. CYP27A1 the enzyme required to introduce the carboxylic acid group at C_27_ is expressed in neurons, oligodendrocytes and some astrocytes in human brain, and in AD brain its expression is reduced in neurons but increased in oligodendrocytes, perhaps reflecting a decrease and increase in their respective cell numbers [46]. CYP3A4 is expressed in neurons, primarily localized in the soma and axonal hillock [45] and CYP7B1 is also expressed in neurons [49]. Yau et al. have shown that in AD brain the per neuron levels of *CYP7B1* mRNA are reduced in hippocampul sections, suggesting a selective impairment in ability of AD brain to 7α-hydroxylate oxysterols [49]. Conversely, HSD3B7 is not expressed in neurons, only glia [48]. From the above we suggest that the reduced level of 7α,25-dihydroxy-3-oxocholest-4-en-26-oic acid in AD CSF is a consequence of a reduced synthesis, resulting from a loss of neuron numbers. Of the cholestenoic acids monitored in this work, only 7α,25-dihydroxy-3-oxocholest-4-en-26-oic acid was found to change in abundance in any patient-group, this suggests that the enzyme responsible for 25-hydroxylation, either CH25H or CYP3A4, is critically important for this effect. *CH25H* is expressed in human cerebral cortex and hippocamps inclusive of pyramidal neurons [48], [50]. *CH25H* is located on chromosome 10, close to the *LIPA* (lipase A, lysosomal acid type) gene which codes for a lysosomal protein which hydrolyses cholesterol esters, in a region strongly linked to AD [51]. Haplotypes in the 5′ untranslated region of *CH25H* are associated with the risk of AD [52], [53]. Whatever the pathway of formation, we can only speculate whether reduced synthesis of 7α,25-dihydroxy-3-oxocholest-4-en-26-oic acid directly contributes to the pathophysiology of AD, as very little is known of the biological activities of this molecule.

How 7α,25-dihydroxy-3-oxocholest-4-en-26-oic acid is metabolised is not known. It may fall into the Duane bile acid biosynthesis pathway [54] with formation of 7α,25-dihydroxy-3,24-*bis*oxocholest-4-en-26-oic acid and elimination of 2-oxopropanoic acid with formation of 7α-hydroxy-3-oxochol-4-en-24-oic acid and ultimately chenodeoxycholic acid or be reduced at Δ^4^ by AKR1D1 (aldo-keto reductase family 1 member D1) then at C-3 by AKR1C4 (aldo-keto reductase family 1 member C4) prior to C-24 carbonylation and elimination of 2-oxopropanoic acid (Fig. 7). Although AKR1D1 and 1C4 are usually regarded as liver specific, Mano et al. have shown that enzymes in the cytosolic fraction prepared from rat brain can convert 7α-hydroxy-3-oxochol-4-en-24-oic acid to chenodeoxycholic acid [55]. Whether these enzymes are present in human brain and can similarly act on C_27_ acids is unknown. Further studies of CSF targeting additional metabolites are required to learn more about the metabolism of 7α,25-dihydroxy-3-oxocholest-4-en-26-oic acid.

The authentic standard of 3β,7α,25-trihydroxycholest-5-en-26-oic acid, from which 7α,25-dihydroxy-3-oxocholest-4-en-26-oic acid is synthetically derived by bacterial cholesterol oxidase treatment, was only recently custom synthesised by Avanti Polar Lipids Inc. We await synthesis of an isotope-labelled version to fully validate the current findings on a larger cohort of AD patients.

Previous studies have shown that the concentration of 7α,25-dihydroxy-3-oxocholest-4-en-26-oic acid in CSF is higher than in serum [9], and we suggest that in the healthy brain 7α,25-dihydroxy-3-oxocholest-4-en-26-oic acid provides a route for removal of cholesterol via metabolism to more a hydrophilic metabolite. Its reduced concentration in CSF of the AD patient-group suggests that this route is attenuated in this disease state. It cannot be ruled out that, like many other cholesterol-derived molecules, 7α,25-dihydroxy-3-oxocholest-4-en-26-oic acid may also have signalling properties which become diminished in the disease state. In light of the reduced level of 7α,25-dihydroxy-3-oxocholest-4-en-26-oic acid in CSF of the AD patient-group it is tempting to devise routes to enhance its formation. Although perhaps a naïve suggestion, if CYP3A4 is the necessary 25-hydroxylase this could be up-regulated through activation of the pregnane X receptor (PXR, NR1I2) or the constitutive androstane receptor (CAR, NR1I3), as CYP3A4 is a target gene of both PXR and CAR [56], [57]. PXR is activated by multiple agonists including dexamethasone, rifampicin and enzyme-inducing antiepileptics, as well as many constituents of herbal remedies, while CAR is activated by several environmental chemicals and pharmaceuticals [58], [59], [60]. In fact, the effects in human of PXR activation by rifampicin, also known as rifampin, which demonstrates good CNS penetration [61], have recently been assessed in a randomized, open, placebo-controlled crossover trial on an oral glucose tolerance test [62]. The PXR agonists elicited postprandial hyperglycemia, suggesting a detrimental role of PXR activation on glucose tolerance. Although none of the participants reached the clinical criterion of impaired glucose tolerance [62], there are obvious limitations of this approach. Alternatively, cholesterol 25-hydroxylase, CH25H, an interferon-stimulated gene, may be activated via the interferon receptor [63]. Interestingly, Merck KGaA have performed a clinical trial (ClinicalTrials.gov Identifier: NCT01075763) to evaluate interferon β-1a in the treatment of AD.

In summary, we have identified a statistically significant reduction of 7α,25-dihydroxy-3-oxocholest-4-en-26-oic acid in the CSF of the patient-group with AD. Based on LC-MS identification of cholesterol metabolites and correlations of their concentrations, we suggest mechanisms for the formation of 7α,25-dihydroxy-3-oxocholest-4-en-26-oic acid. Interestingly, one of the suggested pathways relies on *CH25H*, an interferon stimulated gene implicated in AD.

## Appendix A Supplementary material

Supplementary material Supplemental Figure S1. Principals of EADSA technology illustrated by 3β,7α-dihydroxycholest-5-en-(25 R)26-oic and 7α-hydroxy-3-oxocholest-4-en-(25 R)26-oic acids derivatised with [^2^H_5_]GP and [^2^H_0_]GP, respectively.

Supplementary material Supplemental Figure S2. Dot-plots displaying the concentrations of different oxysterols in CSF. Each dot indicates an individual patient sample. Concentrations are in ng/mL. The black bars indicate the mean value for each group. There were no statistical differences between the disease and control groups. (A) 24S-HC, 24S-hydroxycholesterol; (B) 25-HC, 25-hydroxycholesterol; (C) (25 R)26-HC, (25 R)26-hydroxycholesterol; (D) 25D_3_, 25-hydroxyvitamin D_3_; (E) 7α-HC, 7α-hydroxycholesterol; (F) 7β-HC, 7β-hydroxycholesterol; (G) 7O-C, 7-oxocholesterol. Abbreviations: - AD, Alzheimer's disease; VD, vascular dementia; OND, other neurodegenerative diseases, i.e. Lewy bodies dementia, Frontotemporal dementia. Supplemental Figure S3. The acidic pathway of bile acid biosynthesis. Metabolites from the neutral pathway (shown in blue box) can enter the acidic pathway after C-26 hydroxylation and carboxylation. Coloured bars indicate metabolites detected but not changed in concentration. Red, green and blue correspond to Alzheimer's disease, vascular dementia or other neurodegenerative disease, respectively. Abbreviations: - ACOX2, acyl-coenzyme A oxidase 2; AKR, aldo-keto reductase; AMACR, α-methylacyl-CoA racemase; BACS, bile acid-CoA synthetase; CYP, Cytochrome P450; DBP, D-Bifunctional protein; HSD3B7, 3β-hydroxysteroid dehydrogenase type 7; SPCx, Sterol carrier protein x; VLCS, Very long chain Co-A synthetase. Supplemental Figure S4. Dot-plots displaying the concentrations of different dihydroxysterols and cholestenoic acids in CSF. Each dot indicates an individual patient sample. Concentrations are in ng/mL. The black bar indicates the mean value. (A) 7α,25-diHCO, 7α,25-dihydroxycholest-4-en-3-one; (B) 7α,(25 R)26-diHCO, 7α,(25 R)26-dihydroxycholest-4-en-3-one; (C) 3β-HCA, 3β-hydroxycholest-5-en-(25 R)26-oic acid; (D) 3O-CA, 3-oxocholest-4-en-(25 R)26-oic acid; (E) 3β,7α-diHCA, 3β,7α-dihydroxycholest-5-en-(25 R)26-oic acid; (F) 7αH,3O-CA, 7α-hydroxy-3-oxocholest-4-en-(25 R)26-oic acid; (G) 3β,7β-diHCA, 3β,7β-dihydroxycholest-5-en-(25 R)26-oic acid. Supplemental Figure S5. Dot-plots displaying the concentrations of different dihydroxy-3-oxocholest-4-en-26-oic and chol-4-en-24-oic acids in CSF. Each dot indicates an individual patient sample. Concentrations are in ng/mL. The black bar indicates the mean value. Only 7α,25-dihydroxy-3-oxocholest-4-en-26-oic acid showed a statistical difference between the disease and control groups. (A) 7αH,3O-Δ^4^-BA, 7α-hydroxy-3-oxochol-4-en-24-oic acid; (B) 7αH-26-nor-C-3,24-diO, 7α-hydroxy-26-*nor*-cholest-4-en-3,24-dione; (C) 7α,24-diH,3O-CA, 7α,24-dihydroxy-3-oxocholest-4-en-26-oic acid; (D) 7α,x-diH,3O-CA, 7α,x-dihydroxy-3-oxocholest-4-en-26-oic acid; (E) 7α,25-diH,3O-CA, 7α,25-dihydroxy-3-oxocholest-4-en-26-oic acid; (F) 7α,12α-diH,3O-CA, 7α,12α-dihydroxy-3-oxocholest-4-en-26-oic acid. 7α-Hydroxy-26-*nor*-cholest-4-en-3,24-dione is formed by decarboxylation of 7α-hydroxy-3,24-*bis*oxocholest-4-en-26-oic acid [8]. The location of the second hydroxy group, indicated by x, in 7α,x-diH,3O-CA is on the C_17_ side-chain, probably at C-22 or C-23 [12]. Supplemental Figure S6. Dot-plots displaying the concentrations of a cholestatrien-3β-ol, cholesterol, desmosterol and 8-dehydrocholestrerol in CSF. Each dot indicates an individual patient sample. Concentrations are in ng/mL. The black bar indicates the mean value. (A) Triene, cholestatrien-3β-ol; (B) Cholesterol; (C) Des, desmosterol; (D) 8-DHC, 8-dehydrocholesterol.

Supplementary material Supplemental Figure S7. Biosynthesis of 7α,25-dihydroxy-3-oxocholest-4-en-26-oic acid in the CNS starting with 7α-hydroxylation- (CYP7A1) or (25 R)26-hydroxylation- (CYP27A1) of cholesterol. Coloured bars indicate metabolites detected but not changed in concentration, while coloured arrows indicate metabolites changed in concentration. Red, green and blue correspond to Alzheimer's disease, vascular dementia or other neurodegenerative disease, respectively. Metabolites, named by red text show significant Spearman's rank correlation coefficients to 7α,25-dihydroxy-3-oxocholest-4-en-26-oic acid. Abbreviations: CYP, cytochrome P450; HSD, hydroxysteroid dehydrogenase.

Supplementary material Supplemental Table S1. Demographics of the patients studied.

Supplementary material Supplemental Table S2. Concentration (ng/mL) of the different oxysterols and bile acid precursors measured in CSF from the different patient-groups.

Supplementary material Supplemental Table S3. Concentration (ng/µg-cholesterol) of the different oxysterols and bile acid precursors measured in CSF from the different patient-groups.

Supplementary material Supplemental Table S4. Concentrations of cholesterol and its precursors measured in CSF from different patient-groups.
