# Supplementary figures and images for: Concentrations of bile acid precursors in cerebrospinal fluid of Alzheimer's disease patients
Source: Free Radic Biol Med. 2019 Apr;134:42–52. doi: 10.1016/j.freeradbiomed.2018.12.020 (PMC6597949; doi:10.1016/j.freeradbiomed.2018.12.020)

## Slide 1
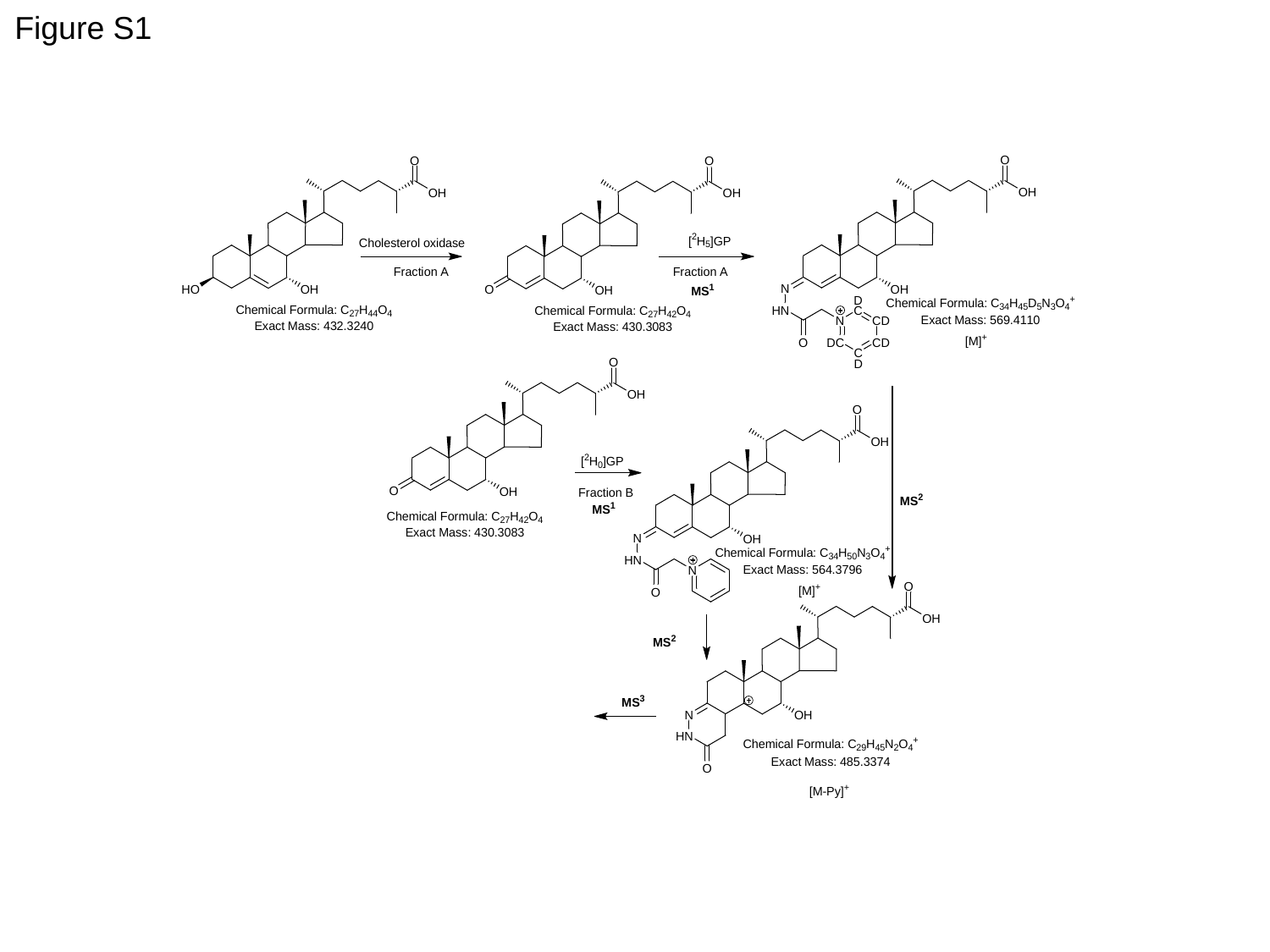

Figure S1

Supplement: Supplementary file 1 — Supplementary material Supplemental Figure S1. Principals of EADSA technology illustrated by 3β,7α-dihydroxycholest-5-en-(25 R)26-oic and 7α-hydroxy-3-oxocholest-4-en-(25 R)26-oic acids derivatised with [2H5]GP and [2H0]GP, respectively. [file mmc1.pptx]

## Slide 1
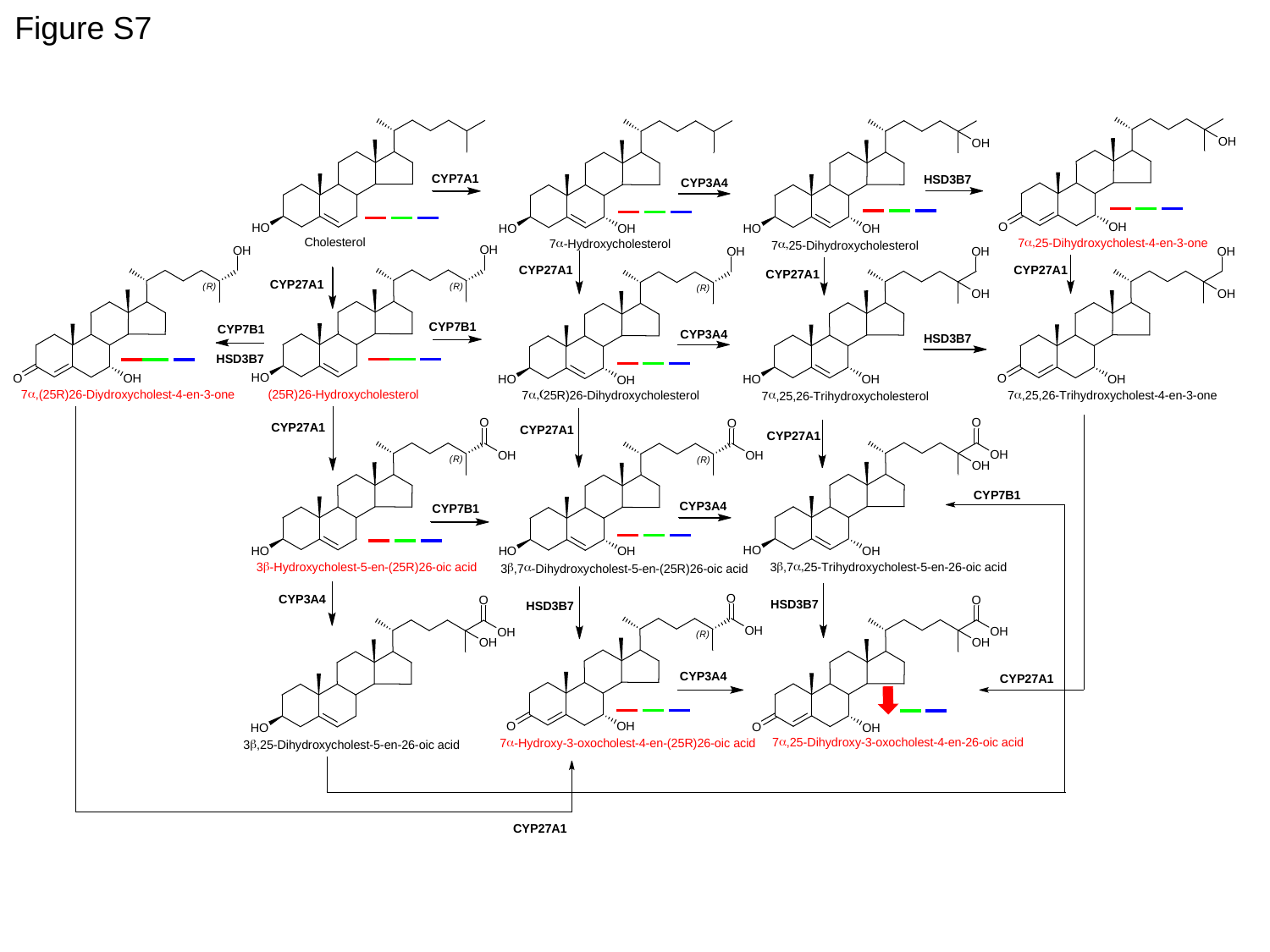

Figure S7

Supplement: Supplementary file 3 — Supplementary material Supplemental Figure S7. Biosynthesis of 7α,25-dihydroxy-3-oxocholest-4-en-26-oic acid in the CNS starting with 7α-hydroxylation- (CYP7A1) or (25 R)26-hydroxylation- (CYP27A1) of cholesterol. Coloured bars indicate metabolites detected but not changed in concentration, while coloured arrows indicate metabolites changed in concentration. Red, green and blue correspond to Alzheimer's disease, vascular dementia or other neurodegenerative disease, respectively. Metabolites, named by red text show significant Spearman's rank correlation coefficients to 7α,25-dihydroxy-3-oxocholest-4-en-26-oic acid. Abbreviations: CYP, cytochrome P450; HSD, hydroxysteroid dehydrogenase. [file mmc3.pptx]
