# Supplementary material for: Concentrations of bile acid precursors in cerebrospinal fluid of Alzheimer's disease patients
Source: Free Radic Biol Med. 2019 Apr;134:42–52. doi: 10.1016/j.freeradbiomed.2018.12.020 (PMC6597949; doi:10.1016/j.freeradbiomed.2018.12.020)
Supplement: Supplementary file 2 — Supplementary material Supplemental Figure S2. Dot-plots displaying the concentrations of different oxysterols in CSF. Each dot indicates an individual patient sample. Concentrations are in ng/mL. The black bars indicate the mean value for each group. There were no statistical differences between the disease and control groups. (A) 24S-HC, 24S-hydroxycholesterol; (B) 25-HC, 25-hydroxycholesterol; (C) (25 R)26-HC, (25 R)26-hydroxycholesterol; (D) 25D3, 25-hydroxyvitamin D3; (E) 7α-HC, 7α-hydroxycholesterol; (F) 7β-HC, 7β-hydroxycholesterol; (G) 7O-C, 7-oxocholesterol. Abbreviations: - AD, Alzheimer's disease; VD, vascular dementia; OND, other neurodegenerative diseases, i.e. Lewy bodies dementia, Frontotemporal dementia. Supplemental Figure S3. The acidic pathway of bile acid biosynthesis. Metabolites from the neutral pathway (shown in blue box) can enter the acidic pathway after C-26 hydroxylation and carboxylation. Coloured bars indicate metabolites detected but not changed in concentration. Red, green and blue correspond to Alzheimer's disease, vascular dementia or other neurodegenerative disease, respectively. Abbreviations: - ACOX2, acyl-coenzyme A oxidase 2; AKR, aldo-keto reductase; AMACR, α-methylacyl-CoA racemase; BACS, bile acid-CoA synthetase; CYP, Cytochrome P450; DBP, D-Bifunctional protein; HSD3B7, 3β-hydroxysteroid dehydrogenase type 7; SPCx, Sterol carrier protein x; VLCS, Very long chain Co-A synthetase. Supplemental Figure S4. Dot-plots displaying the concentrations of different dihydroxysterols and cholestenoic acids in CSF. Each dot indicates an individual patient sample. Concentrations are in ng/mL. The black bar indicates the mean value. (A) 7α,25-diHCO, 7α,25-dihydroxycholest-4-en-3-one; (B) 7α,(25 R)26-diHCO, 7α,(25 R)26-dihydroxycholest-4-en-3-one; (C) 3β-HCA, 3β-hydroxycholest-5-en-(25 R)26-oic acid; (D) 3O-CA, 3-oxocholest-4-en-(25 R)26-oic acid; (E) 3β,7α-diHCA, 3β,7α-dihydroxycholest-5-en-(25 R)26-oic acid; (F) 7αH,3O-CA, 7α-hydroxy-3-oxo [file mmc2.pptx]

## Slide 1
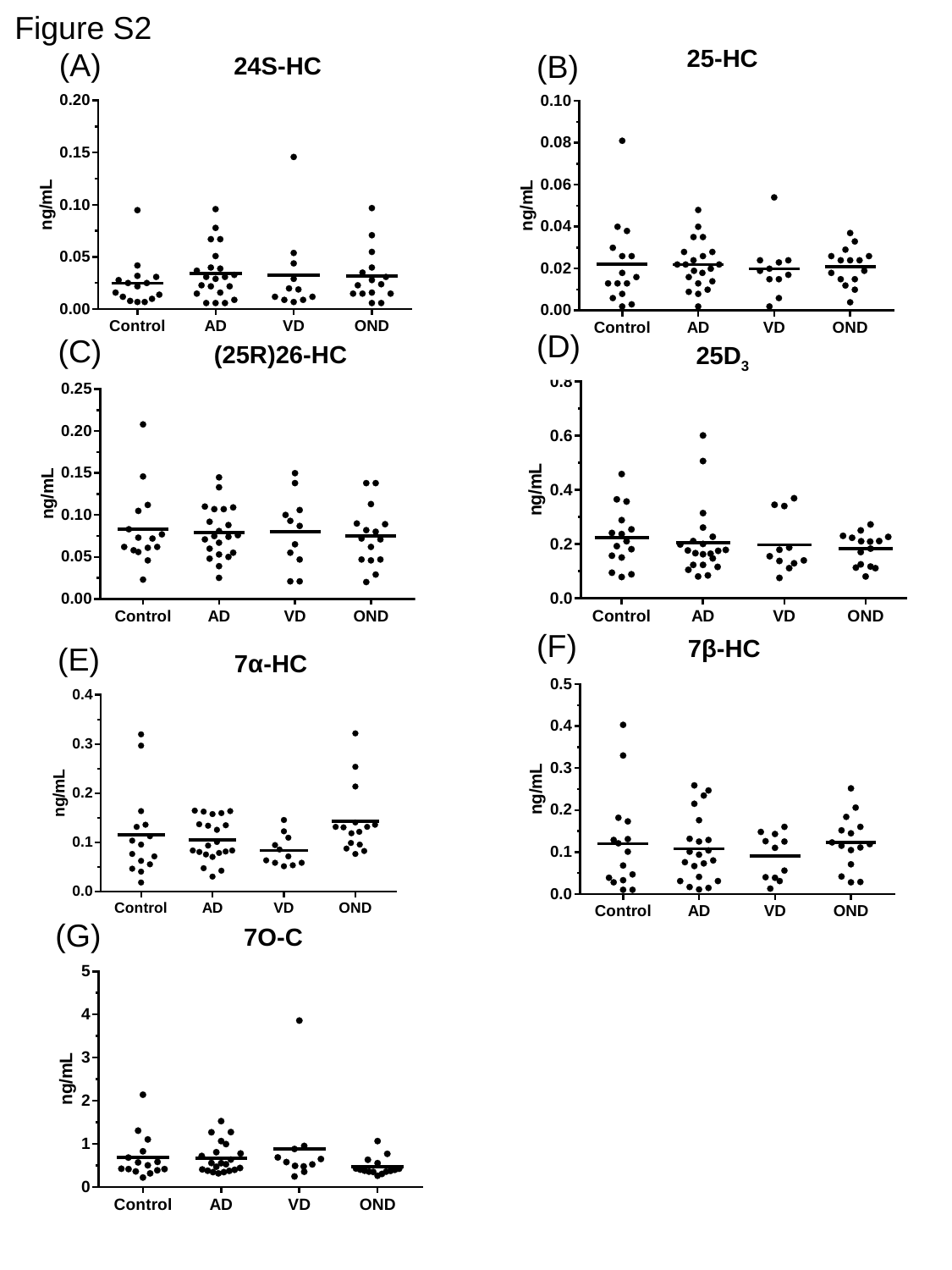

Figure S2
25-HC
(A)
(B)
24S-HC
(D)
(C)
(25R)26-HC
25D3
(F)
7β-HC
(E)
7α-HC
(G)
7O-C

## Slide 2
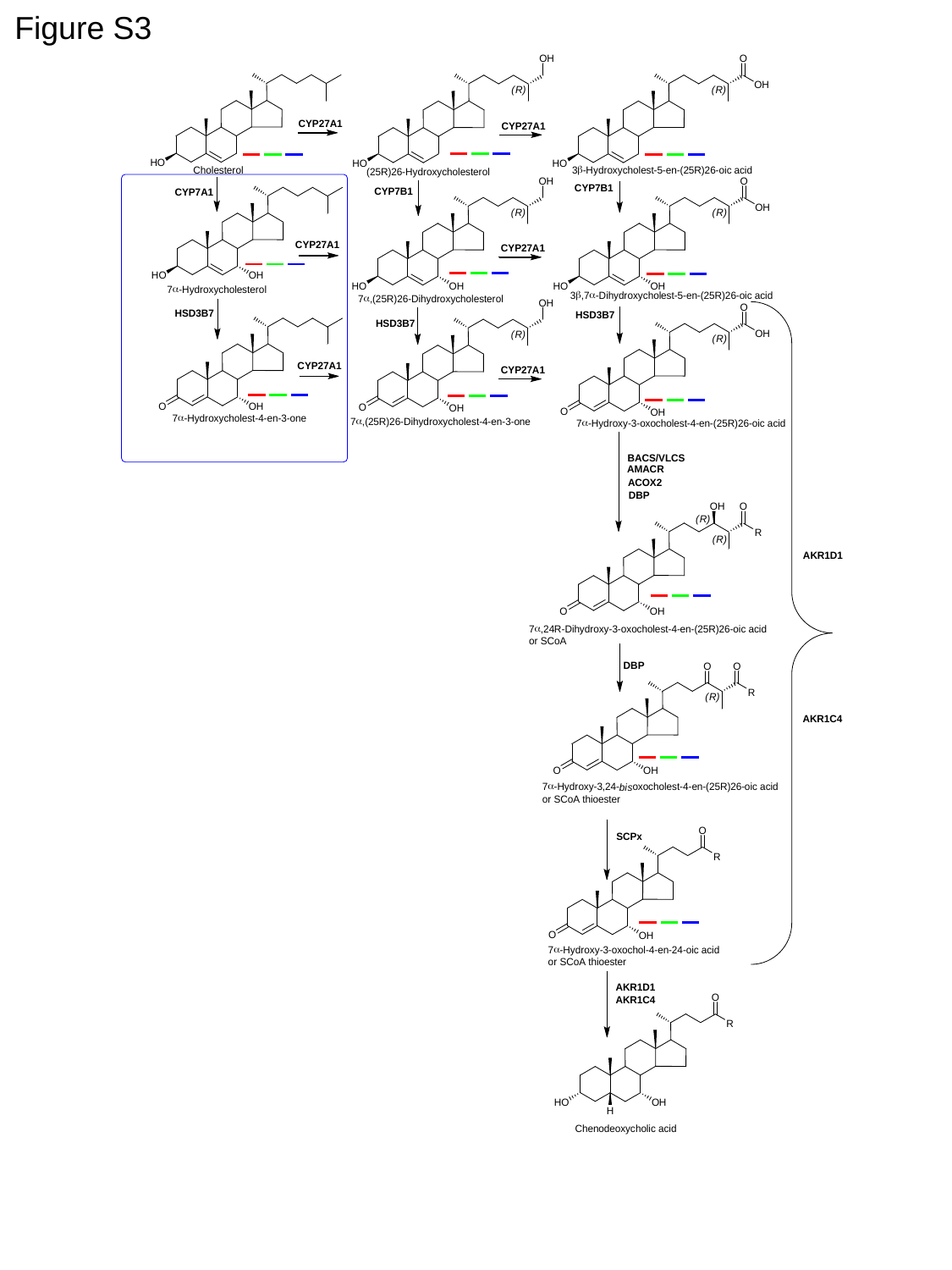

Figure S3
#

## Slide 3
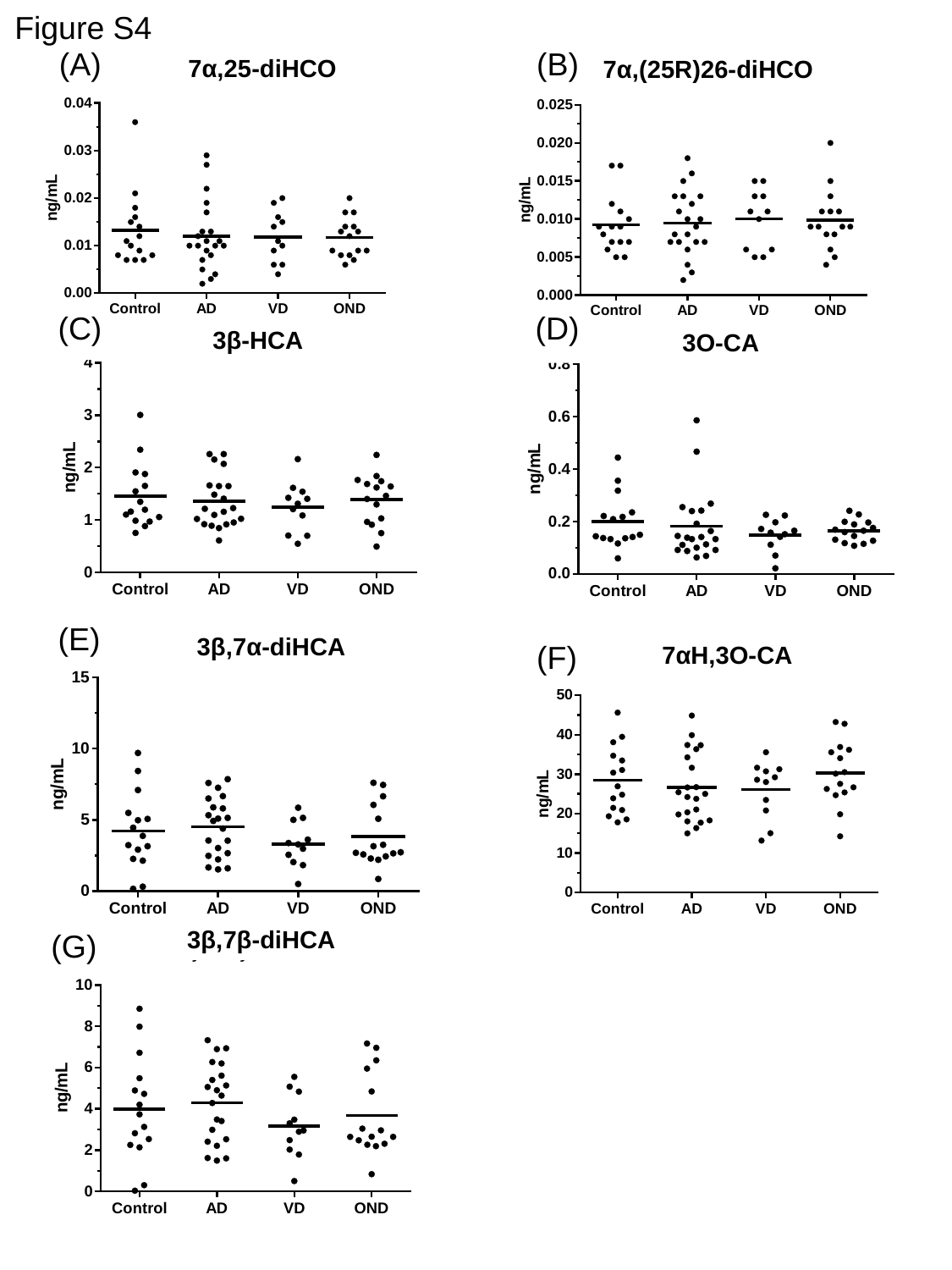

Figure S4
(A)
(B)
7α,25-diHCO
7α,(25R)26-diHCO
(C)
(D)
3β-HCA
3O-CA
(E)
3β,7α-diHCA
(F)
7αH,3O-CA
3β,7β-diHCA
(G)

## Slide 4
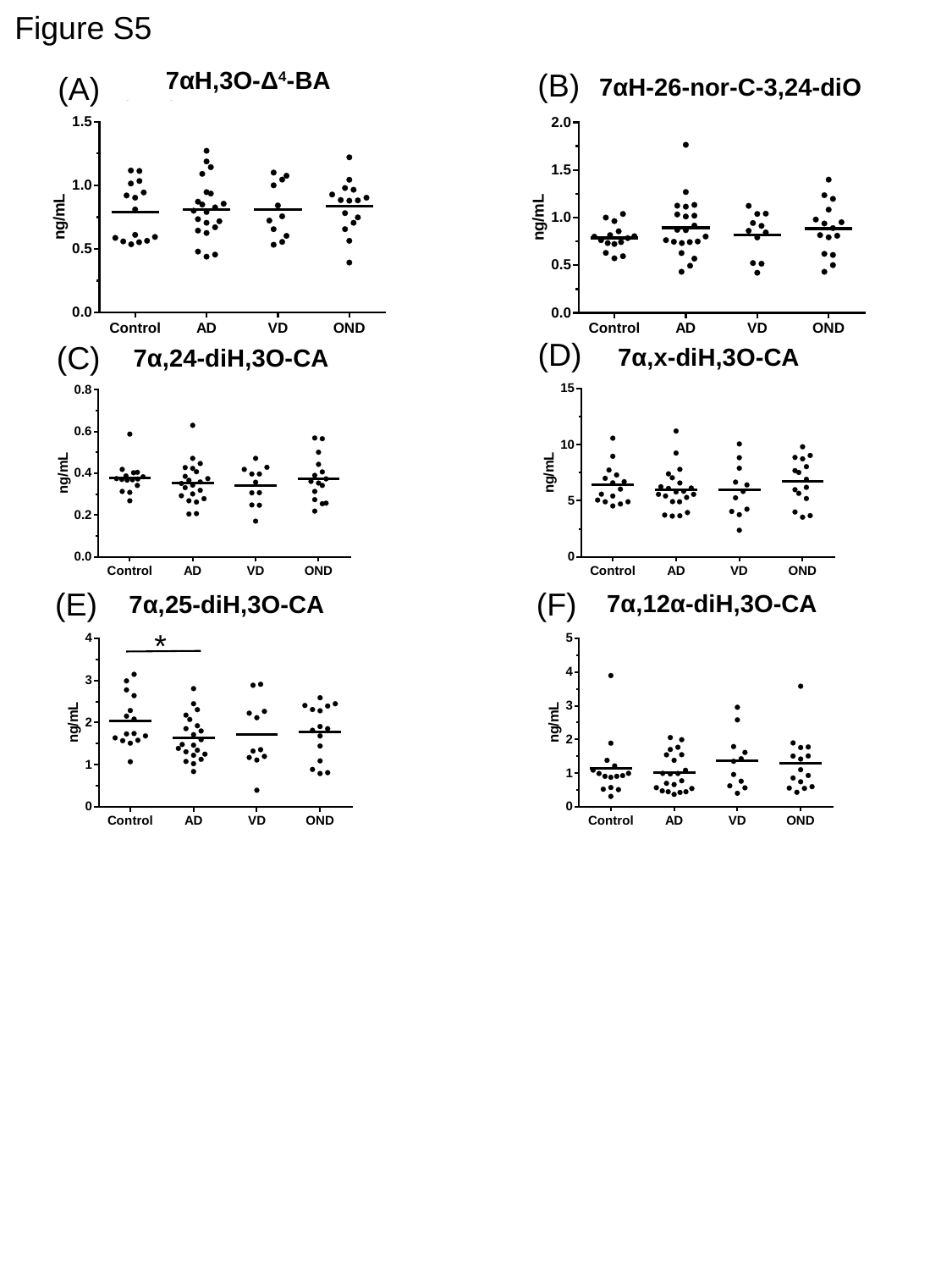

Figure S5
7αH,3O-Δ4-BA
(B)
(A)
7αH-26-nor-C-3,24-diO
(D)
(C)
7α,x-diH,3O-CA
7α,24-diH,3O-CA
(E)
(F)
7α,12α-diH,3O-CA
7α,25-diH,3O-CA
*

## Slide 5
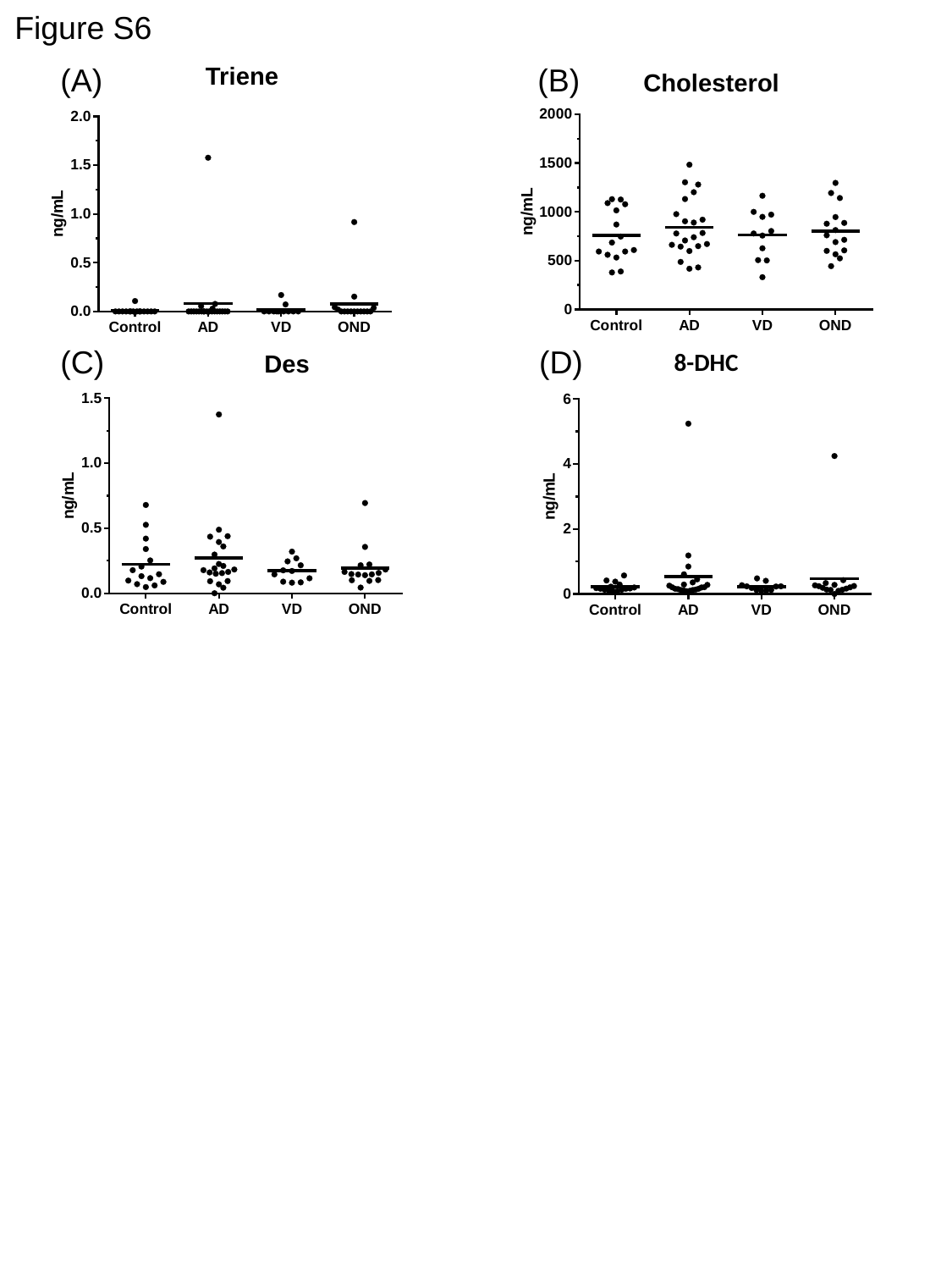

Figure S6
(A)
(B)
Triene
Cholesterol
(C)
(D)
8-DHC
Des
