# Supplementary material for: Concentrations of bile acid precursors in cerebrospinal fluid of Alzheimer's disease patients
Source: Free Radic Biol Med. 2019 Apr;134:42–52. doi: 10.1016/j.freeradbiomed.2018.12.020 (PMC6597949; doi:10.1016/j.freeradbiomed.2018.12.020)
Supplement: Supplementary file 4 — Supplementary material Supplemental Table S1. Demographics of the patients studied. [file mmc4.docx]

Supplemental Table S1. Demographics of the patients studied.

| **Random Code** | **Age**  **Sex** | **Diagnosis** |
| --- | --- | --- |
| 730934 | 75/F | Alzheimer disease FDG-PET-confirmed |
| 657154 | 79/M | Lewy bodies dementia |
| 180673 | 88/F | Alzheimer disease FDG-PET-confirmed |
| 297945 | 73/M | Normal Control (No evidence of NPH) |
| 443835 | 70/M | Normal Control (No evidence of NPH) |
| 411299 | 78/F | Alzheimer disease + NPH |
| 826921 | 79/F | Frontotemporal dementia |
| 477583 | 75/M | Frontotemporal dementia (Primary Progressive Aphasia PET) |
| 883004 | 76/M | Alzheimer disease Brain-biopsy-confirmed |
| 187536 | 76/M | Alzheimer disease |
| 715065 | 69/F | Alzheimer disease Brain-biopsy-confirmed |
| 466797 | 76/M | Normal Control (No evidence of NPH) |
| 473898 | 61/F | Alzheimer disease FDG-PET-confirmed + NPH |
| 876707 | 67/M | Alzheimer disease Biopsy-confirmed + NPH |
| 986866 | 74/F | Frontotemporal dementia |
| 484872 | 81/F | Vascular Dementia |
| 815829 | 73/F | Frontotemporal dementia + NPH |
| 841903 | 64/F | Normal Control - Depression |
| 467513 | 75/F | Alzheimer disease + NPH |
| 760942 | 93/M | Alzheimer disease + NPH |
| 597359 | 68/M | Lewy bodies dementia |
| 375637 | 81/F | Normal Control (No evidence of NPH) |
| 993783 | 62/F | Frontotemporal dementia |
| 925705 | 77/F | Frontotemporal dementia |
| 943568 | 85/F | Vascular Dementia |
| 216052 | 76/F | Alzheimer disease + NPH |
| 454537 | 86/M | Vascular dementia |
| 433961 | 59/F | Normal control (No evidence of NPH) |
| 628435 | 79/M | Frontotemporal dementia + NPH |
| 808558 | 74/M | Vascular dementia |
| 800896 | 78/M | Vascular dementia |
| 464469 | 81/F | Alzheimer disease FDG-PET-confirmed |
| 713114 | 79/M | Normal control (No evidence of NPH) |
| 311280 | 80/M | Alzheimer disease Biopsy-confirmed + NPH |
| 369255 | 74/M | Alzheimer disease FDG-PET-confirmed |
| 641423 | 73/M | Normal control (No evidence of NPH) |
| 758158 | 77/M | Normal control (No evidence of NPH) |
| 401092 | 83/M | Normal control (No evidence of NPH) |
| 282237 | 80/M | Normal control (No evidence of NPH) |
| 581939 | 57/F | Vascular dementia |
| 425543 | 87/M | Alzheimer disease + NPH |
| 508239 | 68/F | Dementia ONS |
| 612950 | 76/M | Normal control (No evidence of NPH) |
| 727275 | 80/F | Lewy bodies dementia |
| 780203 | 75/F | Alzheimer disease |
| 662645 | 90/M | Vascular dementia |
| 713772 | 71/M | Vascular dementia |
| 253902 | 65/M | Frontotemporal dementia |
| 867856 | 64/F | Alzheimer disease |
| 285145 | 71/F | Alzheimer disease |
| 137395 | 81/M | Normal control (No evidence of, NPH) |
| 786665 | 79/M | Frontotemporal dementia + NPH |
| 693749 | 63/M | Frontotemporal dementia |
| 739477 | 84/M | Normal control (No evidence of, NPH) |
| 611808 | 64/M | Frontotemporal dementia + NPH |
| 759694 | 81/F | Vascular dementia + NPH |
| 147296 | 76/F | Alzheimer disease |
| 668532 | 64/M | Normal control (No evidence of NPH) |
| 689994 | 64/M | Alzheimer disease |
| 617196 | 70/M | Alzheimer disease |
| 853566 | 77/M | Vascular dementia |
| 261544 | 69/M | Vascular dementia |

**SUMMARY**

| Alzheimer disease | N=21 | N=4 confirmed by brain biopsy |
| --- | --- | --- |
| Other neurodegenerative disease | N=15 | LBD (3), FTD (11) |
| Vascular dementia | N=11 | Not mixed with AD |
| Normal controls | N=15 | No evidence of NPH |
|  |  |  |
| TOTAL | N=62 subjects |  |
